# Durability of Wood–Cement Composites with Modified Composition by Limestone and Stabilised Spruce Chips

**DOI:** 10.3390/ma17246300

**Published:** 2024-12-23

**Authors:** Tomáš Melichar, Amos Dufka, Karel Dvořák, Patrik Bayer, Silvestr Vasas, Iveta Novakova, Ivana Schwarzova, Jiří Bydžovský

**Affiliations:** 1 Institute of Technology of Building Materials and Components, Faculty of Civil Engineering, Brno University of Technology, 602 00 Brno, Czech Republic; amos.dufka@vut.cz (A.D.); karel.dvorak@vut.cz (K.D.); xvasas00@vut.cz (S.V.); jiri.bydzovsky@vut.cz (J.B.); 2 Institute of Chemistry, Faculty of Civil Engineering, Brno University of Technology, 602 00 Brno, Czech Republic; patrik.bayer@vut.cz; 3 Department of Building, Energy and Material Technology, Faculty of Engineering Science and Technology, The Arctic University of Norway, 8514 Narvik, Norway; iveta.novakova@uit.no; 4 Department of Material Engineering, Institute of Environmental Engineering, Faculty of Civil Engineering, Technical University of Kosice, 042 00 Kosice, Slovakia; schwarzova.ivana@dagslovakia.sk

**Keywords:** wood–cement composite, particleboard, limestone, by-product, cuttings, secondary spruce chips, stabilisation, long-term durability, mechanical properties, microstructure

## Abstract

Limestone (LS) and stabilised secondary spruce chips (SCs) utilisation in wood–cement composites is still an unexplored area. Therefore, the main objective of the research presented here is the assessment of the long-term behaviour of cement-bonded particleboards (CBPs) modified by LS and SCs. Cement (CE) was replaced by 10% of LS, and spruce chips by 7% of SCs. The test specimens were stored in a laboratory and exterior environment (Middle Europe) for up to 2 years. The density, strength, and modulus of elasticity were evaluated after 28 days, and then in 6-month periods. The hygroscopicity was analysed separately. The mineralogical composition and microstructure were analysed due to possible LS participation during hydration. SC synergic behaviour in CBPs was also studied. After 2 years, the microstructure of the CBP was more compact, and denser. Strong carbonatation contributes to the improvement of CBP properties. The products of carbonatation were present in both the matrix and wood chips. The hydration of the matrix was almost finished. LS has a positive effect on the matrix microstructure development. LS acts both as an active component participating in the formation of the cement matrix structure and as an inert microfiller, synergic with hydration products. SCs have a positive effect on the hygroscopic behaviour of CBPs and slightly negative effect on the tensile strength.

## 1. Introduction

Cement-bonded particleboards remain a relatively widespread and popular material, as evidenced by their global production and numerous applications. In Europe, the production of these boards takes place in several countries, including the Czech Republic (CIDEM Hranice, a.s.), Germany (Eternit, Binos, Amroc), Hungary (Falco Wood Industry), the Netherlands (Eltomation), Turkey (Betopan), Portugal (Viroc), Italy (Investwood), and Belarus (CSP BZS). The composition of these wood–cement composites varies by manufacturer, typically containing approximately 50–65% cement by mass and 18–25% wood chips. The remainder of the mix consists of water and hydration or stabilisation additives. Considering, for example, the annual production of CIDEM Hranice, a.s., which is 55,000 m^3^ [1,2] or about 78,000 t (calculated according to the density of CBP), the cement consumption for this manufacturer alone amounts to approximately 39,000 t per year (estimated according to CBP composition). This is not an insignificant amount, given the need for substantial quantities of mineral resources, energy (with cement firing temperatures of at least 1450 °C), and the resultant CO_2_ emissions. Therefore, it is evident that when summing up the raw material requirements of all the mentioned manufacturers, the reported cement consumption for the production of cement-bonded particleboards is significantly higher. Thus, the possibility of substituting cement with suitable alternative raw materials presents itself. One such option is micronized limestone.

The topic of replacing cement and the potential activities of limestone, i.e., its involvement in hydration reactions or its role in actively shaping a compact cement matrix, remains a subject of research. The conclusions of various authors, i.e., whether it acts as an active ingredient or merely as an inert filler, are not entirely conclusive, and differ. The relevance of the issue at hand has been evidenced by many publications for few decades [3,4,5]. Rahnal et al. [6] admits both the direct and non-direct stimulation of Portland cement hydration when these effects are intensified as the cement replacement ratio increases. The addition of limestone can provide a filler effect for hydration acceleration, and also react with dissolved aluminates to form carboaluminate phases, such as monocarbonate and hemicarbonate [7,8,9]. The decrease in Hemicarbonate and increase in monocarbonate in time was observed by Shao et al. [10]. Bahulayan and Santhanam [11] proved that blends of CE with low-grade limestone show an acceleration in early hydration, similar to the acceleration shown by blends with pure limestone. Oey et al. [12] characterised LS as a mineral additive rather than a mineral filler. The reason is that LS has the ability to serve as a preferred surface and produce chemical (ion sorption) effects. This indicates LS’s ability to serve as more than just a filler in cementing systems. Wang et al. [13] proved that with the increase of LS content in concrete, the thickness of the ITZ decreases first, and then increases. The density of the ITZ and pore structure of the paste in concrete is optimal at an LS content of 10%. Wang et al. [14] found a positive synergistic effect of LS and rice husk ash (RHA) at a content of 10% for both components. The acceleration of C_3_S hydration is presented among others in [15,16,17,18]. Despite different opinions of LS acting in cementitious materials, practically all authors agree that optimally chosen amounts of limestone lead to improved properties of cement composites. Similarly, many scientists are aware that the field requires further detailed research to clarify and characterise the phenomena associated with the formation of a cement matrix modified by finely ground limestone.

The interaction between limestone and cement, or hydration products, will occur in the presence of Al_2_(SO_4_)_3_·18H_2_O (AS), in the case of wood–cement composites. This substance is used as an accelerator for the hydration process. Therefore, this represents another variable from the perspective of the role of limestone in hydration reactions. AS participates in the hydration reactions and directly influences their progression and the final properties of the matrix [19,20], specifically of the resulting composite. The interaction of AS has been demonstrated during the hydration of a cement matrix containing finely ground limestone [21].

From a long-term perspective, the interaction of limestone in wood–cement composites has not been extensively explored. Finely ground limestone primarily serves as an inert filler in wood–cement composites over shorter time frames [22,23].

**Figure 1:**
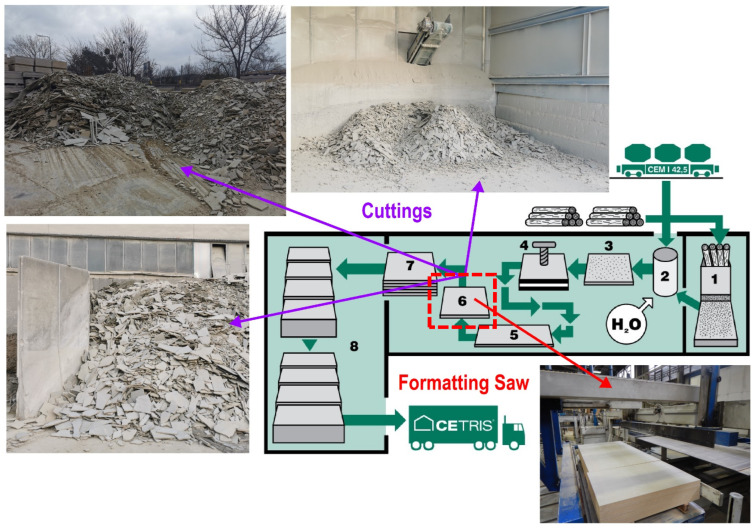
Production scheme of CIDEM Hranice, a.s., particleboards: 1—spilling; 2—preparation of mixture; 3—layering of boards; 4—pressing; 5—drying; 6—formatting; 7—storage; and 8—transport (highlighted cuttings by-product from formatting particleboards) [30].

During the production and processing of cement-bonded particle boards, a significant amount of by-products in the form of dust, cuttings, and particle mixtures are generated. These are still the subject of research and have not yet found further applications. Approximately 5000 t per year of cuttings from the processing of boards (see Figure 1—production scheme of CBP with highlight of cuttings origin) are produced by CIDEM Hranice, a.s.

It has been found that these cuttings can be relatively easily mechanically processed [24,25] to obtain, among other things, fairly high-quality secondary chips with stabilised properties [26]. These can be used as substitutes for primary chips, up to an amount of 7%. The use of secondarily obtained stabilised spruce chips (or particles containing stabilised chips) ensures an improvement—a reduction in dimensional or volumetric changes due to variations in temperature and humidity [26,27]. The utilisation of by-products from the production and processing of wood–cement composites has also been studied by Ezersky et al. [28] and Adelusi et al. [29]. However, these scientists did not explore the reuse of this by-product for the production of CBP.

A review of expert publications revealed that none of the authors had explored the possibility of modifying the composition of cement particle boards using limestone as a substitute for cement. Nor was the possibility of reusing stabilised chips from the production of cement particle boards analysed from a long-term perspective. There were also no findings regarding the research on wood–cement composites of modified composition that had been exposed to real exterior climatic conditions over the long term. It is not known that boards containing limestone and secondary chips have been produced on an industrial scale.

For these reasons, the intent of the research presented here was to thoroughly analyse the properties and microstructure of wood–cement composites in the form of CBP after two years. Emphasis was placed on comparing the behaviour of boards stored long-term in the laboratory and those exposed to the outdoor environment (climatic conditions of the Czech Republic).

## 2. Materials and Methods

Three formulations were compiled (see Table 1), based on the standard recipe (CBP-R) for the production of cement particle boards by CIDEM Hranice, a.s. The reference formulation was modified with finely ground (micronized) limestone (LS) and additionally with secondary stabilised chips (SCs). Thus, the compositions of the CBP-L and CBP-S formulations were defined. In the CBP-L formulation, 10% of the primary binder (CE) was substituted. The composition of the CBP-S formulation was modified more significantly. The substitution amounted to 10% of the binder LS and 7% of the spruce chips SCs, simultaneously. The amount of LS up to 10% was chosen based on previous results [22,23,26] as well as [3,31]. The correctness of the choice is also confirmed by the recent findings presented by Wang et al. [15,32].

### 2.1. Materials

**Figure 2:**
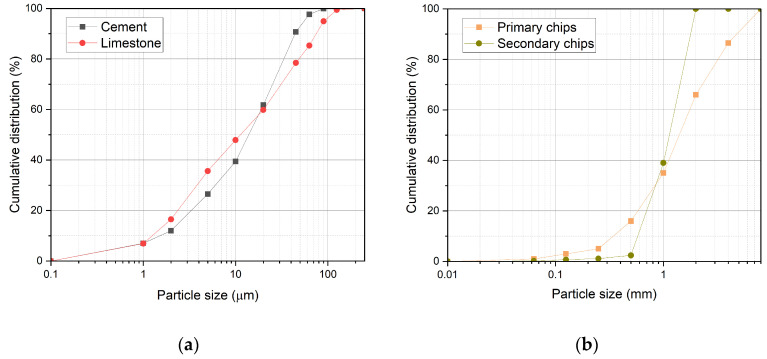
Particle size and distribution: (**a**) cement CEM II/A-S 42.5 R and limestone VMV15-F; (**b**) primary spruce chips and secondary stabilised chips 0.5–2 mm.

The size and distribution of limestone particles are similar to those of cement (see Figure 2a). However, it is noticeable that the limestone contains a slightly higher percentage of grains smaller than 20 µm. Therefore, the limestone used can be characterised as a finer particulate matter, which corresponds with a specific surface area of 476 m^2^/kg (LS) and 408 m^2^/kg (CE; see Table 2). Some authors [32] use LS of smaller grains (ca up to 10 µm). A similar particle size distribution of LS is presented in [13,33,34,35]. In the case of LS’s active contribution to the matrix structure formation, this is a positive finding, as the smaller particle size positively contributes to the potential activity of LS [7]. On the other hand, LS could increase the water/cement ratio of cement paste [36].

Primary spruce chips contain particles up to 8 mm (see Figure 2b). The dominant fraction is 1–2 mm (approximately 31%), followed by the 2–4 mm fraction (approximately 21%) and the 0.5–1 mm fraction (approximately 19%).

For the modification of the board formula, a mixture of secondary chips consisting of two fractions, namely 0.5–1 mm and 1–2 mm in a 1:2 ratio, was prepared. These two fractions were deliberately selected based on previous results [24], which show the highest content of spruce wood. Secondary chips (stabilised spruce wood) contain particles with a maximum size of up to 2 mm. This represents a significant difference in particle size compared to primary chips, which could negatively affect the bending strength [37,38,39,40]. The incorporation of large particles has been found to impact greater bending properties in wood–cement composites; however, poor interfacial bonding between large particles and cement often results in low-strength properties [41] and affects the composite durability [37,42]. Therefore, 7% of primary chips were replaced, also considering previous results [26].

In comparison with cement, limestone is characterised by a lower density (see Table 2). On the other hand, it is a particulate matter with a higher specific surface area. The density of limestone VMV15-F is slightly higher than that reported, for example, by Černý et al. [43] for limestone VBS 40, and slightly lower than the LS used by Kang et al. and Ladjel et al. [15,44]. Secondary chips exhibit very significantly different physical properties compared to primary chips (see Table 2). The bulk density reaches approximately three times that (SC > PC). In the case of the loose bulk mass, this difference is even greater. Radically different values were also determined in the case of the water absorption of SCs and PCs. Here, the stabilisation of SC properties by cement, Na_2_SiO_3_, and Al_2_(SO_4_)_3_·18H_2_O was evident.

From the perspective of chemical composition (see Table 3), cement predominantly contains CaO, SiO_2_, and Al_2_O_3_. The cement used thus exhibits a standard representation of individual components. Approximately 95% of the limestone is composed of CaCO_3_ with a minor proportion of other components (SiO_2_, Al_2_O_3_, etc.). Considering the composition of limestone VMV15-F, a relatively high purity of this raw material is evident [4,11,45,46,47]. The chemical analysis in the case of SCs was focused both on the content of wood (determined from the Total Organic Carbon—TOC) and on the quantity of other components originating from the cement matrix. Residues of this matrix partially cover the surface of the SCs.

Cement CEM II/A-S 42.5 R contains clinker minerals characteristic of Portland clinker, including a setting regulator, as well as phases that occur in blast furnace slag (see Table 4). Limestone predominantly contains calcite, followed by aragonite, and trace amounts of magnesite and quartz. The mineralogical composition corresponds with the results of the chemical analysis (see Table 3).

**Figure 3:**
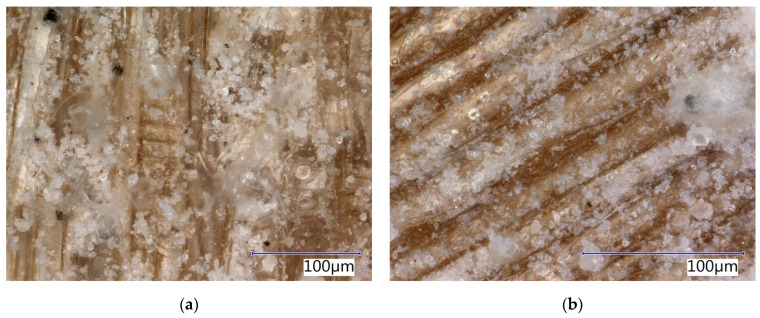
Detail of structure (Keyence VHX-950F optical microscope): (**a**) secondary chips 0.5–1 mm; (**b**) secondary chips 1–2 mm.

**Figure 4:**
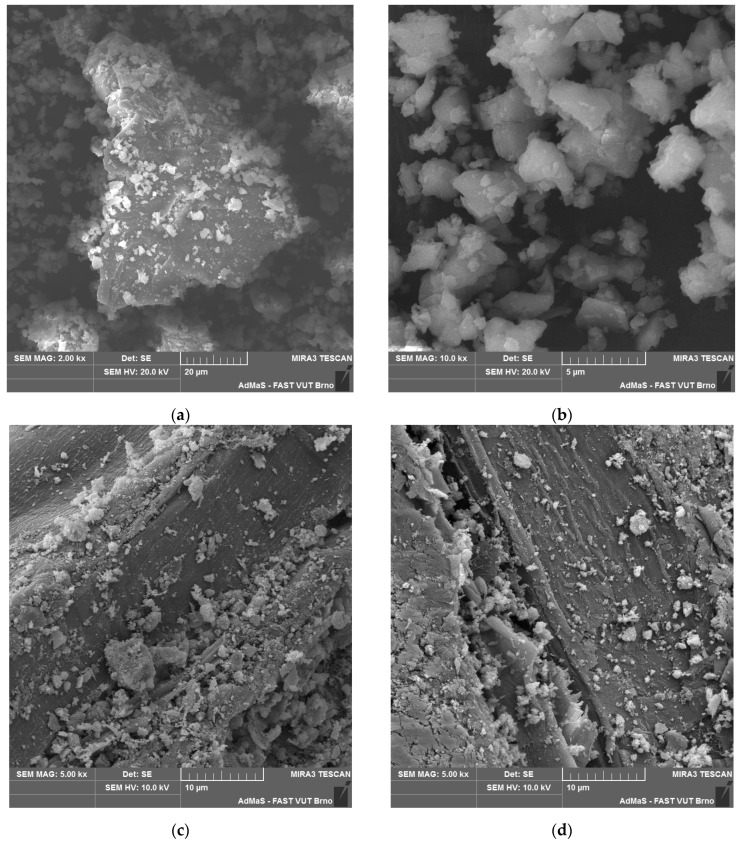
Detail of structure (Tescan MIRA3 XMU scanning electron microscope): (**a**) finely ground limestone; (**b**) finely ground limestone; (**c**) secondary chips 0.5–1 mm; (**d**) secondary chips 1–2 mm.

Phases characteristic of the cement matrix were identified in the case of SCs (see Table 4). These secondary chips are stabilised, and residues of the cement matrix can be identified on their surface (see Figure 3 and Figure 4). Matrix products also penetrate the cellular structure of the spruce chips. This determines the different properties of SCs compared to PCs (see Table 2).

The analysis of the microstructure (see Figure 4c,d) indicates a relatively compact and undamaged structure of spruce chips with residues of the cement matrix. Hydration products, specifically ions from the matrix, have partially penetrated the cellular structure of the spruce wood.

According to the particle size and distribution analysis, limestone contains the most particles in the sizes of 5–10 µm (19.1%) and 20–45 µm (18.6%). These types of LS particles are captured in the SEM images (see Figure 4a,b).

### 2.2. Samples and Curing

**Figure 5:**
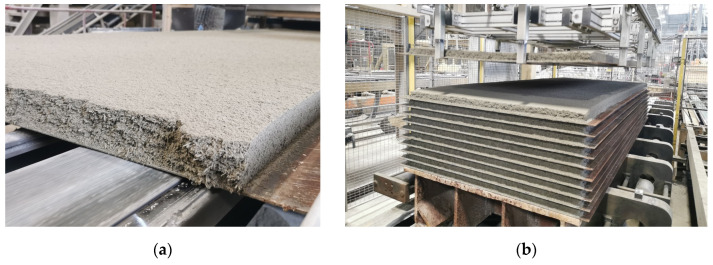
Production of cement-bonded particleboards—CIDEM Hranice, a.s.: (**a**) layered mixture on a steel pad; (**b**) layering of individual steel plates with the mixture over each other prior to pressing.

**Figure 6:**
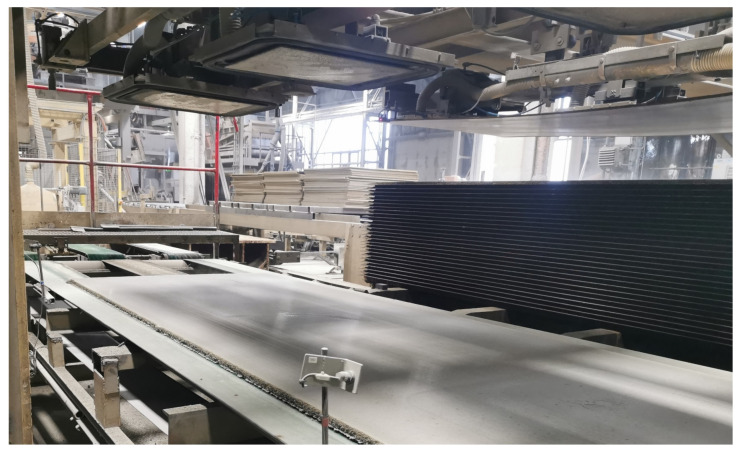
Production of cement-bonded particleboards CIDEM Hranice, a.s.: board after pressing and thermal treatment, before formatting and grinding.

CBPs were manufactured using the standard procedure at the production plant of CIDEM Hranice, a.s. (see Figure 5 and Figure 6). Both reference and modified composition boards were produced to minimise the potential variability from the manual laboratory production in small batches. The complete production scheme is shown in Figure 1. Secondary chips and limestone were manually fed into the mixing equipment. Other components were weighed and dosed automatically as part of the standard production procedure. In the following images (see Figure 5 and Figure 6), no negative phenomena (e.g., the disruption of mixture homogeneity, development of cracks, loss of cohesion during handling of the boards after the heating phase, etc.) were observed during the production of the boards with a modified composition (see Figure 5a). From this perspective, the composition modification can thus be considered successful.

Given the continuity of the production process, it was necessary to produce boards of the given formulation always with a minimum volume of 4 m^3^ (i.e., the volume of approximately four mixing devices). This is where the length of the production line becomes relevant, thus allowing a smooth transition or change in the composition of the boards. To ensure boards of the desired composition, samples were taken from boards produced in the 2nd and 3rd mixing cycles.

**Figure 7:**
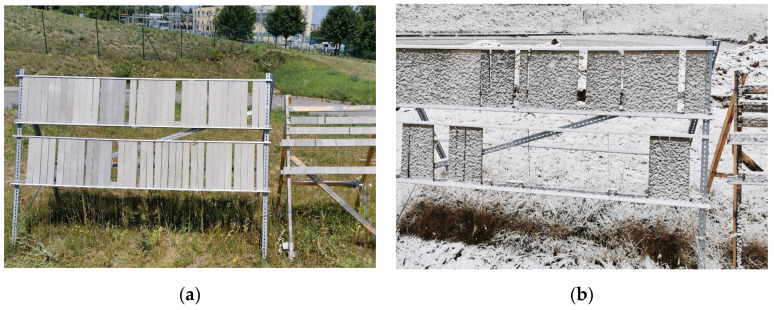
Exposure of test specimens in real climatic conditions over the course of 2 years (Central Europe, Czech Republic): (**a**) summer 2022; (**b**) winter 2023/2024.

From the manufactured boards, test specimens were subsequently created to determine their properties (density, strength, etc.) and to analyse their composition, including the microstructure. A set of 6 test specimens was always used in order to determine each parameter for every formulation and exposure environment. Therefore, 108 test specimens were produced for the analysis of boards from one formulation (totalling 324 pieces). The intent was to assess the impact of the real exposure environment. Half of the specimens (162 pieces) were stored under laboratory conditions at a temperature of (20 ± 2) °C and relative humidity (75 ± 5)%. The other half (162 pieces) were exposed to the real climatic conditions of the Czech Republic (location: AdMaS Center, Purkyňova 139, 612 00 Brno, Czech Republic; see Figure 7)—GPS coordinates: 49°14′08.3″ N 16°34′14.2″ E. The face of the test specimens was oriented southward. An additional 18 test specimens of modified dimensions were made to assess the hygroscopicity. The assessment of the hygroscopicity did not reflect the influence of storing the test specimens in real climatic conditions.

**Figure 8:**
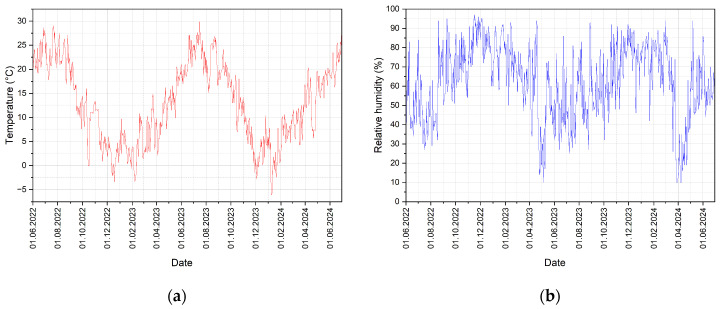
Exposure of test specimens in real climatic conditions over the course of 2 years: (**a**) progression of average daily temperatures; (**b**) progressions of average daily air humidity.

The progression of the average daily temperature and relative humidity under real climatic conditions during the experiment is evident from the following graphs (see Figure 8).

The variations in temperature and humidity are diverse, which is characteristic of the climatic conditions in the Czech Republic (Central Europe). The minimum temperature recorded during the period was −6.1 °C, and the maximum was 29.9 °C. Over the entire two-year monitoring period, the relative humidity of the air ranged from 10% to 97%. Sudden temperature changes (difference between two consecutive days) ranged from −9.8 °C/day to 7.3 °C/day. Fluctuations in relative humidity were recorded in the range of −36%/day to 47%/day. These are not fundamentally significant fluctuations in temperature and humidity. However, wood–cement composites, due to their content of spruce wood, are more susceptible to moisture absorption or desorption, thereby changing their properties, including their structure. Although the changes are not drastic, they can have a rather significant impact on the behaviour of cement particle boards.

### 2.3. Methods and Procedures

During the aging process, the test specimens of wood–cement composites (cement particle boards) were subjected to incremental testing at various time intervals (28, 183, 365, 548, and 730 days). Both the physical and mechanical properties were evaluated, as well as the mineralogical composition, including the microstructure, supplemented by elemental analysis.

Before determining each parameter, the specimens were always maintained (air-conditioned) under defined conditions according to the relevant technical standards [48,49,50,51,52].

The density was determined in accordance with the requirements of the technical standard EN 323 [50]. For this measurement, adjustable digital callipers with base lengths of 150 mm (Mitutoyo 500-181-30) and 400 mm (TIGRE) with a measurement accuracy of 0.01 mm, and scales (Radwag—PS 6000. R1) with an accuracy of 0.01 g were used.

The strength and modulus of elasticity in bending were determined in accordance with the requirements of the technical standard EN 310 [48]. The test setup corresponds to a three-point bending arrangement. The tensile strength perpendicular to the plane of the board was determined according to the provisions of the technical standard EN 319 [49]. A Testometric M350-20CT machine with a 20 kN load cell and an accuracy of ±0.5% was used.

The assessment of hygroscopicity was conducted using a modified method according to EN 318 [52]. This parameter was the only one not evaluated at various ages due to the time-consuming nature of the test. The procedure specified in EN 318 was modified to obtain more detailed information about the absorption and desorption of the tested materials. Specifically, the procedure was carried out incrementally (from 0%) in 10% steps up to a relative humidity of 96%. This was followed by a gradual decrease again in 10% humidity steps down to 0%, i.e., the drying of the test specimens in a drying facility. In total, changes in dimensions, specifically volume and weight, were determined at 21 points. To stabilise the properties (weight) at each of these points, it was necessary to maintain specific conditions for several days (10% and 20%) up to weeks (80%, 90%, and 96%). The entire procedure took approximately 9 months, with hygroscopicity testing commencing when the boards were about 1 year old. The reason for this timing sequence was to simultaneously conclude the hygroscopicity tests, from both the laboratory and outdoor exposure, along with the evaluation of all the parameters. To evaluate the course of the sorption isotherms, the dimensions of the test specimens were also modified to 350 mm × 150 mm × 12 mm. This format of the test specimens corresponds to the realistically manufactured boards in a reduced scale. In addition to the adjustable callipers and scales (see density determination according to EN 323), a digital micrometre (Schut Geometrische Meettechniek bv, Groningen, The Netherlands, range 0–25 mm) and dilatometers with a base of 100 mm and 300 mm fitted with digital inclinometers (MarCator 1086 R; Mahr GmbH, Esslingen, Germany) with an accuracy of 0.001 mm were used.

The mineralogical composition was assessed using an X-ray diffractometer, Empyrean Panalytical (PANalytical B.V., Almelo, The Netherlands; CuKα radiation), with an angular resolution of 0.026°. The quantification of the selected phases was carried out on a differential thermal analyser, Mettler Toledo TGA/DSC 1 STAR System (Mettler-Toledo, LLC, Columbus, OH, USA), with a sensitivity of the DSC sensor of 0.1 mW and a temperature resolution of 0.00003 °C. The analysis of the microstructure and, possibly, elemental composition was performed using a scanning electron microscope TESCAN MIRA3 XMU (TESCAN, Brno, Czech Republic) with resolution of 1.2–1.5 nm at 30 kV in SE mode and 2 nm at 30 kV in BSE mode.

For a detailed assessment of the structure, a Keyence VHX-950F optical microscope (Keyence Ltd., Osaka, Japan) was also used, featuring a maximum magnification of 2500× and the use of a LIBS probe for the elemental analysis.

## 3. Results and Discussion

### 3.1. Mechanical Parameters

**Figure 9:**
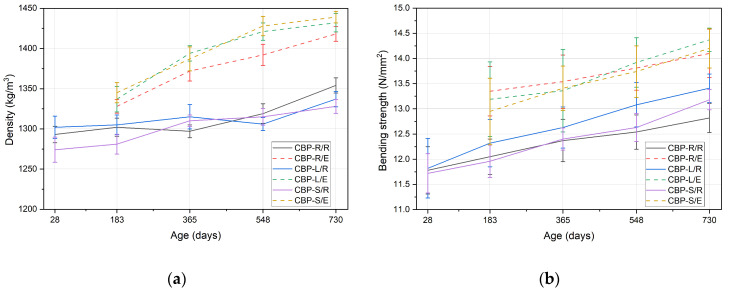
Mechanical parameters of tested CBP: (**a**) density; (**b**) bending strength (exposure: /R—laboratory environment and /E—real climatic conditions).

The density of the tested CBPs (see Figure 9a) ranges from 1274 kg/m^3^ to 1354 kg/m^3^ for boards aged in a laboratory environment. The density gradually increases over time across all tested formulations. Therefore, during aging, there is a progressive development of the matrix structure. A clear dependence of density development on the composition of the boards is not observable. At the end of the monitored period, the highest average density was determined in CBP-R boards. Conversely, CBP-S boards exhibited the lowest density, although the differences are not significant.

Significantly higher density values were found after 730 days exposure in real climatic conditions. This is a significant finding, as despite the influence of adverse environmental conditions, the formation of a more compact matrix, the board structure, still occurred. After 730 days, the boards showed a density range from 1418 kg/m^3^ to 1439 kg/m^3^. Density development curves in the real environment show a steeper increase compared to the trend of density in boards stored in the laboratory. Interestingly, when exposed to real conditions, CBP-L and CBP-S boards exhibit a higher density than the reference boards. The values of the coefficient of variation (see Table 5) indicate a decrease in variability of the monitored parameter over time. While the different exposure environments did impact the variability of the density, a clear dependency cannot be discerned. It is also evident that the achieved values of the coefficient of variation are considerably low. Therefore, it cannot be definitively stated that the influence of real adverse effects leads to an increase or decrease in the variability of individual density values of the boards.

All types of modified boards exhibit a density higher than 1000 kg/m^3^, which is in accordance with the requirements of the technical standard EN 634-2 [51].

The density is lower in comparison with the results [39,53,54,55]. The determined density values can be described as relatively high considering the research outcomes of other authors [40,56,57] who used various alternative raw materials. The density is then slightly higher than that reported by Zhou and Kamdem [58], Maail et al. [59], and Ferrandez-García et al. [60].

The bending strength (see Figure 9b) of boards with the modified composition CBP-L and CBP-S shows a slightly higher increase compared to the reference CBP-R boards. After 28 days of aging in a laboratory environment, the strengths reached 11.7 N/mm^2^ to 11.8 N/mm^2^. These values are very consistent. After 730 days, the strengths ranged between 12.8 N/mm^2^ and 13.4 N/mm^2^. Boards exposed to real climatic conditions exhibited flexural strength in the range of 14.1 N/mm^2^ to 14.4 N/mm^2^, with CBP-L being rated the best. CBP-R and CBP-S boards exhibit similar strengths. The progression of strength curves for the boards exposed in real conditions is interesting. After 183 days of aging, CBP-R achieves the highest strengths, and with increasing age, CBP-L can be rated the best. This development of bending strength indicates the beneficial effect of matrix modification with finely ground limestone.

All types of modified boards exhibit a flexural strength higher than 9.0 N/mm^2^, which is in accordance with the requirements of the technical standard EN 634-2 [51]. At the same time, the determined strength values exceed 11.5 N/mm^2^, which is the average value declared by the manufacturer CIDEM Hranice, a.s.

The determined bending strengths can be evaluated as relatively high in light of the results of relevant research by other authors [37,38,39,40,54,57,61], or higher compared to results [56,58,59] that used various alternative materials. Conversely, the bending strength can be considered lower in comparison with the outputs from Ferrandez-García [55].

The variability of individual flexural strength values (see Table 6) ranges from 1.5% to 5.0% for boards stored in a laboratory environment and from 1.6% to 6.1% for boards aging in real climatic conditions. Compared to the density, these are several-times-higher values. The variability of flexural strength slightly increases due to the influence of the adverse (external) environment, and shows a roughly decreasing trend over time. It is interesting to note that the highest and simultaneously lowest values of the coefficient of variation were determined for CBP-L/E, which could again suggest that limestone progressively participates in the formation of the cement matrix structure. For CBP-S, this effect is not as pronounced. However, in the case of CBP-S, another variable enters the composition of the formulation, which is the substitution of primary chips with secondary chips.

**Figure 10:**
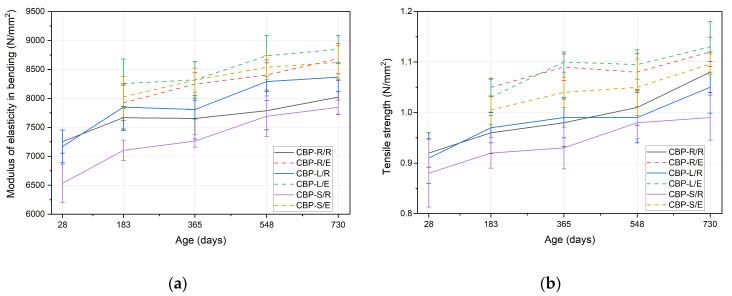
Mechanical parameters of tested CBP: (**a**) modulus of elasticity in bending; (**b**) tensile strength perpendicular to the plane of the board (exposure: /R—laboratory environment and /E—real climatic conditions).

The modulus of elasticity in bending (see Figure 10a) shows an increase with aging similar to the bending strength (see Figure 9b). However, the development trend is not as smooth. The difference between the values determined on boards stored in laboratory and real environments is lower than in the case of bending strength. Boards aging in real climatic conditions exhibit a modulus of elasticity ranging from 8623 N/mm^2^ to 8847 N/mm^2^ after 730 days of exposure. The modification of the composition with secondary chips was quite pronounced. In the laboratory environment, CBP-S boards reach a modulus of elasticity from 6534 N/mm^2^ to 7845 N/mm^2^. In contrast, CBP-L boards show a gradual increase in modulus of elasticity over time from 7171 N/mm^2^ to 8366 N/mm^2^. Given the results obtained, it is evident that the influence of particle size (chips) is more significant here than in the case of bending strength. The modulus of elasticity in bending thus represents a material characteristic that is more sensitive to changes in material composition than bending strength.

All types of modified boards exhibit a modulus of elasticity in bending higher than 4500 N/mm^2^, which is in accordance with the requirements of the technical standard EN 634-2 [51] (for Class 1). The simultaneously determined modulus of elasticity values (except for CBP-S boards) exceeds 6800 N/mm^2^, which is the average value declared by the manufacturer CIDEM Hranice, a.s.

The modulus of elasticity in bending can be considered relatively high in light of the research results of other authors [37,38,39,40,55,57,59,60,61] who used various alternative materials. The determined values are slightly higher than those reported by Yel and Urun [56], for example. A similar modulus of elasticity values was found by Zhou and Kamdem [58].

The coefficients of variation for the modulus of elasticity values (see Table 7) fluctuate due to the age and different compositions of the boards without any apparent dependencies. For example, in the case of CBP-R/R, an increasing trend can be observed. In contrast, CBP-L and CBP-S boards tend to show a more irregular and fluctuating trend of the coefficient of variation.

The tensile strength perpendicular to the plane of the board (see Figure 10b) is also a significant mechanical property. The trends of the tensile strength curves for CBP-R and CBP-L are similar. CBP-S boards exhibit lower tensile strengths. In the case of all formulations and both exposure environments, an increase in strength was observed. Real climatic conditions, as with previous monitored parameters, contributed to the improvement of tensile strength, thus increasing the values. After 730 days of aging, the boards exhibited strengths ranging from 1.10 N/mm^2^ to 1.13 N/mm^2^. In contrast, boards stored in the laboratory showed strengths ranging from 0.99 N/mm^2^ to 1.05 N/mm^2^. Overall, considering the values of tensile strengths, CBP-R boards can be rated the best. This is thus a relatively sensitive indicator of the influence of changes in CBP composition. On the other hand, the differences in tensile strength among the different formulations are very low.

All types of modified boards exhibit a tensile strength perpendicular to the plane of the board higher than 0.5 N/mm^2^, which is in accordance with the requirements of the technical standard EN 634-2 [51]. The simultaneously determined strength values exceed 0.63 N/mm^2^, which is the average value declared by the manufacturer CIDEM Hranice, a.s. The tensile strength can be rated as excellent compared to the research results of other authors [40] who used various alternative materials. The achieved results appear comparable to or lower when compared with values reported by Yel and Urun [56], Zhou and Kamdem [58], and Maail et al. [59]. Ferrandez-García et al. [60] report lower to comparable tensile strengths of boards. Ferrandez-García et al. [55] report a significant dispersion of values depending on the composition of the boards. The tensile strength of boards with a 14% content of jute according to Ferrandez-García et al. [55] approaches the determined values for CBP-R and CBP-L. An interesting finding is that Ferrandez-García et al. [55] recorded a decrease in tensile strength for almost all tested formulations as they aged (from 28 days to 365 days).

The values of the coefficient of variation (see Table 8) show no dependencies or a regular increasing or decreasing trend. Therefore, it is not possible to state whether the variability of this parameter changes depending on the material composition of the CBP, age, or exposure environment.

### 3.2. Hygroscopicity

**Figure 11:**
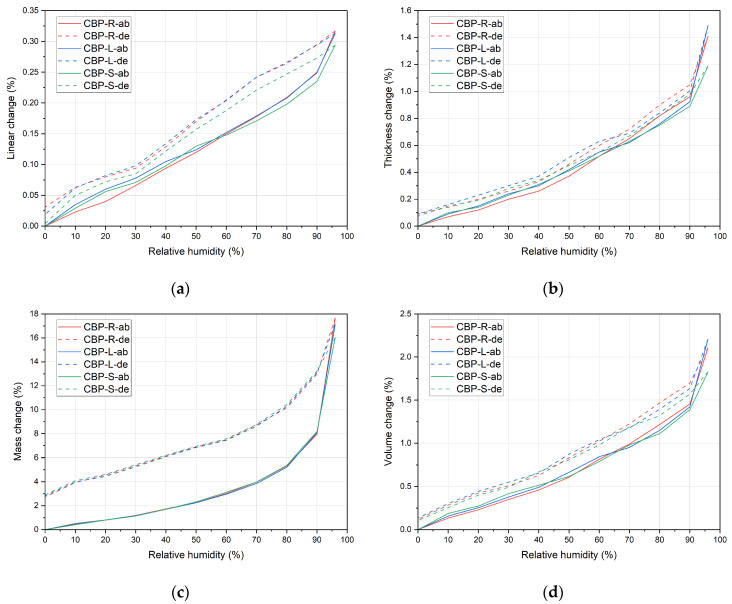
Sorption isotherms with hysteresis effect of tested CBP: (**a**) linear change; (**b**) thickness (lateral) change; (**c**) mass change; and (**d**) volume change (all specimens were stored under laboratory conditions).

In the analysis of the hygroscopic behaviour of the boards, changes in the linear dimensions, thickness, weight, and volume were assessed (see Figure 11). The CBP-S boards are rated the best, showing the smallest changes in these parameters. Practically, in the case of all monitored parameters, the CBP-R, L, and S boards behave similarly. A significant change occurs upon reaching a 90% relative humidity. Between 90% and 96%, the CBP-R and L boards exhibit a steeper increase and ultimately higher changes. This effect is most noticeable in the changes in thickness and volume. Conversely, in terms of the maximum changes in weight (16.0% to 17.7%), very slight differences are observed. Linear changes generally reach the lowest values of maximum changes (0.29% to 0.32%). The absorption isotherms of the evaluated boards practically mirror each other (mass change) or show very slight deviations from each other’s courses. In the range of 65% to 90%, it is noticeable that CBP-L and CBP-S exhibit lower volumetric changes, including the thickness. This could be related to the phenomenon where substituting cement with fine-grained limestone disrupts the continuity of the capillary-porous system (for pores larger than 100 nm). The microstructure of this matrix then becomes more compact [62,63,64], and thus less permeable to water.

The maximum changes and desorption isotherms more distinctly reflect the different compositions of the analysed boards. The different manifestation of hysteresis, particularly in the assessment of linear changes, is also interesting. It is evident that CBP-S boards (containing stabilised secondary chips) are able to return to their original state (linear change 0.04‰), thus essentially representing a reversible phenomenon. The hysteresis effect was most pronounced in the case of changes in weight, where an irreversible change ranging from 2.72% to 2.90% was recorded (see Figure 11c).

The evaluation of dimensional changes according to EN 318 is presented in the following table (see Table 9). The determined values indicate the best resistance of CBP-S boards to changes due to their saturation with water in the gaseous state. Desorption, occurring with a decrease in relative humidity from 65% to 35%, results in a more significant change in length or thickness compared to an increase in humidity from 65% to 85%. This effect is more noticeable in terms of linear changes. During desorption, changes in length were observed to be about 100% higher compared to absorption, while changes in thickness were approximately 35%.

In the context of subsequent research, with regard to favourably influencing, among other things, the hygroscopic behaviour of wood–cement composites, attention will be devoted to the use of the synergistic effect of superabsorbent polymers and alternative silicate components. This subject has been analysed in detail, for example, by Melichar et al. [65].

### 3.3. Phase Composition

**Figure 12:**
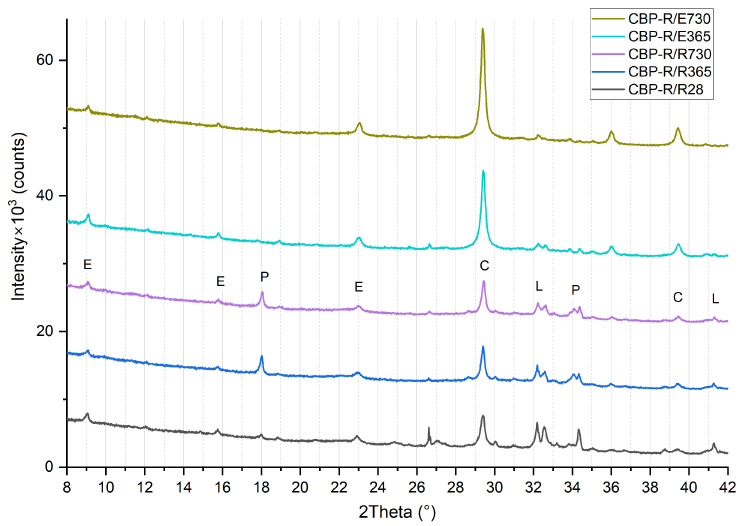
Phase composition of reference wood–cement composite CBP-R—standard industry mixture of company CIDEM Hranice, a.s. (R28, R365, and R730—laboratory environment after 28 days, 365 days, and 730 days, E365 and E730—real climate of Czechia after 365 days and 730 days; E—ettringite, P—portlandite, C—calcite, and L—larnite).

**Figure 13:**
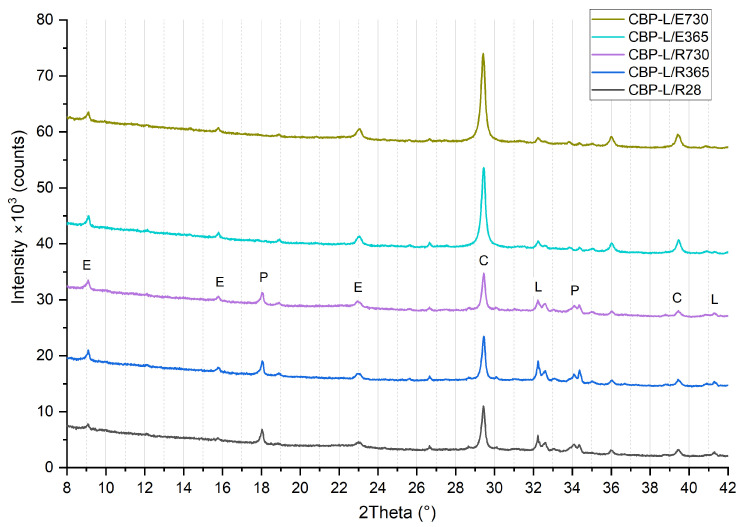
Phase composition of modified wood–cement composite CBP-L—industry mixture modified by limestone (R28, R365, and R730—laboratory environment after 28 days, 365 days, and 730 days, E365 and E730—real climate of Czechia after 365 days and 730 days; E—ettringite, P—portlandite, C—calcite, and L—larnite).

**Figure 14:**
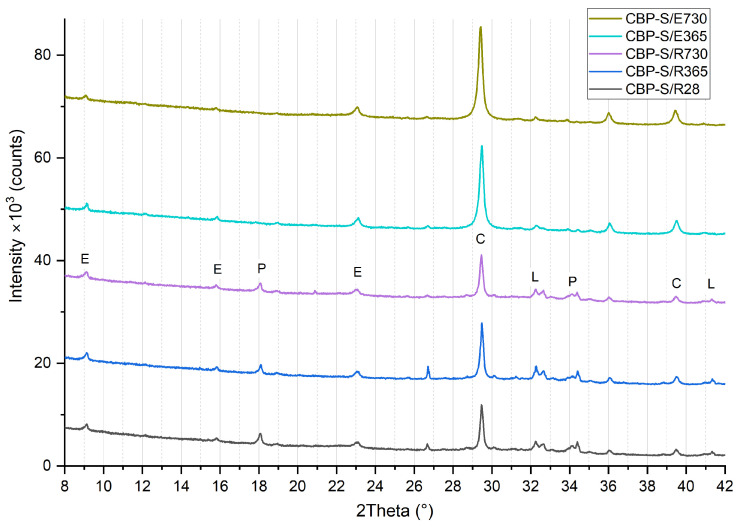
Phase composition of modified wood–cement composite CBP-R—industry mixture modified by limestone and secondary chips (R28, R365, and R730—laboratory environment after 28 days, 365 days, and 730 days, E365 and E730—real climate of Czechia after 365 days and 730 days; E—ettringite, P—portlandite, C—calcite, and L—larnite).

The results of the XRD analysis (see Figure 12, Figure 13 and Figure 14) demonstrate that, compared to storage in a laboratory, significant carbonatation occurred in real climatic conditions. The reaction of Ca(OH)_2_ with CO_2_ to form CaCO_3_ in this case is more dynamic. This was evidenced by a significant increase in the intensity of the diffraction lines characteristic of calcite. At the same time, there is a corresponding decrease in the diffraction lines of portlandite, already evident at 365 days of age. After 730 days of aging, the diffraction lines of calcite reach their highest intensity, and the elimination of portlandite peaks is also apparent. The diffraction lines of portlandite and calcite (except for CBP-R) do not practically change when stored under laboratory conditions.

In real climatic conditions, the presence of SO_2_ must also be considered. A higher amount of fine-grained limestone in the matrix affects the durability of the cement composite. In the presence of SO_2_, these composites are more susceptible to thaumasite formation [66]. Degradation due to thaumasite formation can occur in the cement matrix containing fine-grained limestone (CO_3_^2−^) in the presence of a solution containing (SO_4_^2−^), leading to the disintegration of C–S–H phases in the cement paste (SiO_3_) in the presence of Ca^2+^ [66,67]. This process is accelerated in cold environments with temperatures below 15 °C. However, it is a relatively slow degradation process [68] (also considering the low-to-trace concentration of SO_2_ in the typical atmosphere). Thaumasite formation was not identified in the analysed types of boards. It could possibly be present only in trace amounts, assuming there was an overlap of diffraction lines with ettringite (9.2° and 23.34°). Yang et al. [69] report common diffraction lines for ettringite and thaumasite within an aggressive environment containing sulphur ions. This is a rather complex issue that requires the separate verification of CBP in subsequent research. It would be appropriate to analyse boards stored in real exterior conditions over a longer period (at least 5 years) and simultaneously conduct accelerated laboratory tests (increased temperature, relative humidity, and SO_2_ concentration).

In the analysed materials, ettringite was identified regardless of the formulation composition, exposure environment, and aging period. According to [5], the presence of LS enables the formation of monocarbonate, thereby indirectly stabilising the structure of ettringite at the expense of monocarbonate. This phenomenon has been observed and confirmed by other authors [70,71,72,73]. Hemicarbonate and monocarbonate presence, with hemicarbonate gradual transformation into monocarbonate, was proved by Wang et al. [32]. Confirmation that LS participated in the chemical reaction through the formation of CO^3−^AFm phases and stabilisation of ettringite was presented by Kang et al. [15]. However, monocarbonate was not identified in CBP-R, L, and S, thereby not confirming this effect for the CBP matrix.

The process of gradual long-term hydration can be observed in the change of diffraction lines of larnite (β-C_2_S). During laboratory storage, there is a very gradual change, i.e., a reduction in peak intensity in the region of 32° to 33°. When the boards are stored in real conditions, a significant acceleration of this process and a reduction in intensity almost to background levels are noticeable. It is evident that larnite has practically completely reacted, contributing to the strengthening of the matrix and the cohesion of the entire composite system. Here, a significant correlation is evident with increasing strengths and generally improved properties of boards exposed to real climatic conditions compared to those stored in a laboratory environment. Besides the aforementioned minerals, an amorphous phase—CSH—was also identified. The presence of amorphous CSH around 30° is also confirmed by findings from Liu et al. [74].

Considering the different compositions of the formulations, it is evident that the phase composition of the CBP-R, CBP-L, and CBP-S boards is practically identical. Modifications to the composition with finely ground limestone and stabilised secondary chips did not significantly affect the mineralogical composition (i.e., identified phases). Partial involvement of LS during cement hydration can be inferred from the different progression of portlandite diffraction lines. Compared to CBP-L and CBP-S, CBP-R shows a lower intensity of the Ca(OH)_2_ peak at 18° after 28 days of curing, which corresponds with the findings of Sharma et al. [3]. The intensity of the larnite peaks in the range of 32° to 33° is also different. Boards modified with limestone exhibit lower intensity peaks of β-C_2_S, indicating the accelerated hydration of this clinker mineral [3]. Calcium carboaluminate with a characteristic peak at 13.24° was not identified. The presence of hemicarbonate with a characteristic peak at 10.7° or monocarbonate at 11.7°, as identified Ji et al. [34], was not confirmed. The decreasing intensity of the dominant CaCO_3_ peak (for boards stored in the laboratory) was not recorded, which does not confirm the decomposition and thus the reaction of LS. However, gradual carbonatation can also occur in the laboratory environment, i.e., a gradual increase in the intensity of calcite diffraction lines.

During the quantitative DTA analysis, attention was focused on determining the content of selected phases. The determination was conducted with an emphasis on the following:

- Significant phases concerning the long-term curing of CBP;
- Changes in the content of phases characteristic of the involvement of finely ground limestone in hydration reactions.

DTA results indicate an increasing amount of CaCO_3_ and a corresponding decrease in the content of Ca(OH)_2_ for boards exposed to real climatic conditions. Over time, this carbonatation process leads to the formation of calcite. Considering the different compositions of the formulations, it is significant that CBP-L and CBP-S contain a slightly lower percentage of CSH phases and Ca(OH)_2_. Reducing phases containing chemically bound water in the cement matrix may lead to improved resistance to cyclic effects of freeze and thaw. This phenomenon is confirmed by the results of mechanical parameters (see Figure 9 and Figure 10). The conducted tests did not demonstrate that LS stimulates C-S-H nucleation, giving it a higher efficiency in accelerating clinker hydration [17]. Studying this phenomenon in the matrix of wood–cement composites would require further detailed research in a shorter timeframe. A partial indicator of the active involvement of LS in hydration reactions is the amount of Ca(OH)_2_ formed during hydration. The matrices of CBP-L and CBP-S contain 10% less cement. The Δ values (especially after 28 days; see Table 10) thus demonstrate a higher quantity (relative only to the amount of CE) of Ca(OH)_2_ in the matrices of CBP-L and CBP-S. This finding roughly corresponds with the results of other authors [3], who state that in the presence of LS, cement hydration is accelerated, producing a higher amount of Ca(OH)_2_.

### 3.4. Microstructure

**Figure 15:**
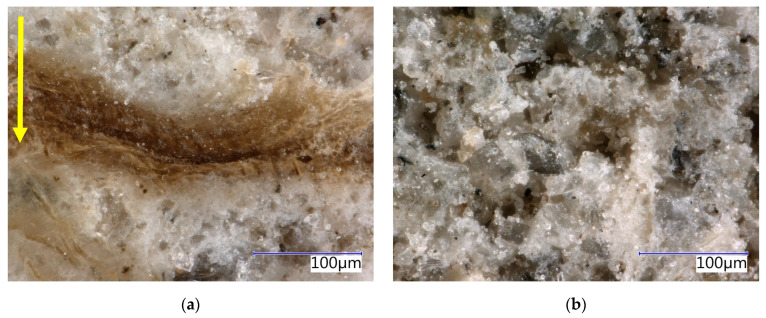
Detail of structure (Keyence VHX-950F optical microscope)—reference boards CBP-R after 730 days ageing in laboratory environment: (**a**) sidewall view, yellow arrow—direction of the board pressing; (**b**) face-side view.

**Figure 16:**
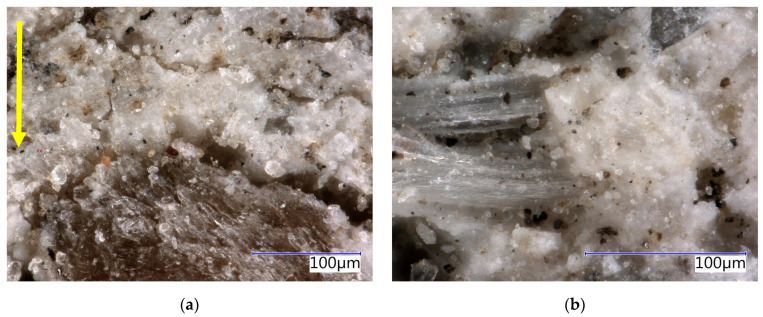
Detail of structure (Keyence VHX-950F optical microscope)—reference boards CBP-R after 730 days ageing in external climatic environment: (**a**) sidewall view, yellow arrow—direction of the board pressing; (**b**) face-side view.

**Figure 17:**
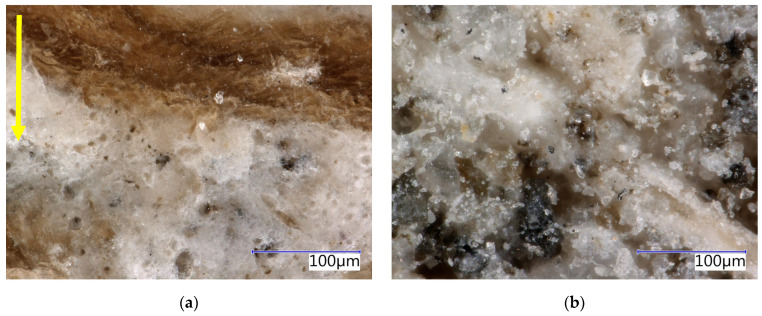
Detail of structure (Keyence VHX-950F optical microscope)—reference boards CBP-L after 730 days ageing in laboratory environment: (**a**) sidewall view, yellow arrow—direction of the board pressing; (**b**) face-side view.

**Figure 18:**
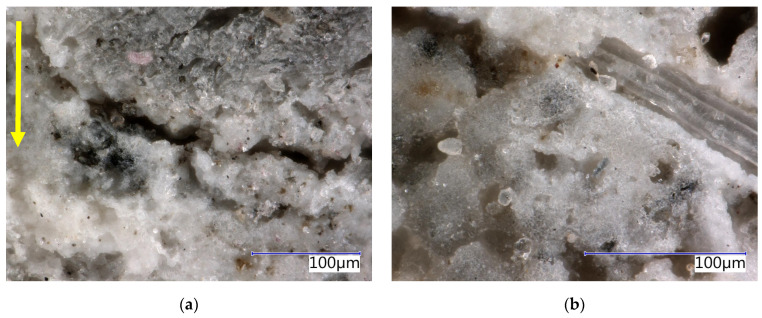
Detail of structure (Keyence VHX-950F optical microscope)—reference boards CBP-L after 730 days ageing in external climatic environment: (**a**) sidewall view, yellow arrow—direction of the board pressing; (**b**) face-side view.

The structure of the boards was analysed in detail using an optical microscope (see Figure 15, Figure 16, Figure 17 and Figure 18). The analysis was conducted on both the front surface and the side of the examined sample. The front surface represents the plane of the board. When the board is applied in real construction, this is the visible surface. The compaction (smoothing) of the boards occurs perpendicular to the front surface. The side characterises the area in the direction of thickness, i.e., parallel to the direction of compaction. The side is therefore perpendicular to the plane of the board. Due to the significant similarity in the structure of CBP-L and CBP-S, only images depicting CBP-L are presented here. In the structure of CBP-S, it was practically impossible to distinguish secondary chips from primary ones.

The images show a compact structure of the wood–cement composites stored in a laboratory environment. The interaction between spruce chips and the cement matrix is in mutual synergy. The continuity of the chips to the cement matrix is smooth. The contact zone between the chips and the matrix is compact and without defects. There is also a noticeable difference in the structure of the side wall and the front surface. A detailed analysis of this surface reveals a continuous porous system.

Test specimens exposed to real environmental conditions exhibit a somewhat different surface structure. Microscopic cracks of varying widths from 5 µm to 40 µm (see Figure 16a) were found. The carbonatation of the matrix is evident from the change in shade of the specimens. It is also noticeable that there has been a change (stabilisation) in the spruce chips. On the front surface, minor defects were locally identified—partial exposure of the chips (see Figure 16b).

The analysis of the surface structure of the wood–cement composites CBP-L (see Figure 17 and Figure 18) and CBP-S with an optical microscope has demonstrated practically identical phenomena as observed in CBP-R. However, the modified materials exhibit a lower range and intensity of disruption due to the adverse conditions of the external environment compared to the reference. The cracks at the interface of the matrix and the chips are less wide open (see Figure 16a and Figure 18a). The matrix structure of the materials modified with limestone appears more compact. This effect can be observed both during laboratory storage and exposure to real climatic conditions. The compactness and cohesion of the matrix after the long-term exposure to weather conditions in CBP-L are evident from Figure 18b.

The cracks identified on the side surface are found predominantly (>90%) at the interface between the matrix and the chips. Occasionally, cracks were also identified within the matrix itself. This phenomenon was slightly more pronounced in CPB-R and the least observable in CBP-S.

**Figure 19:**
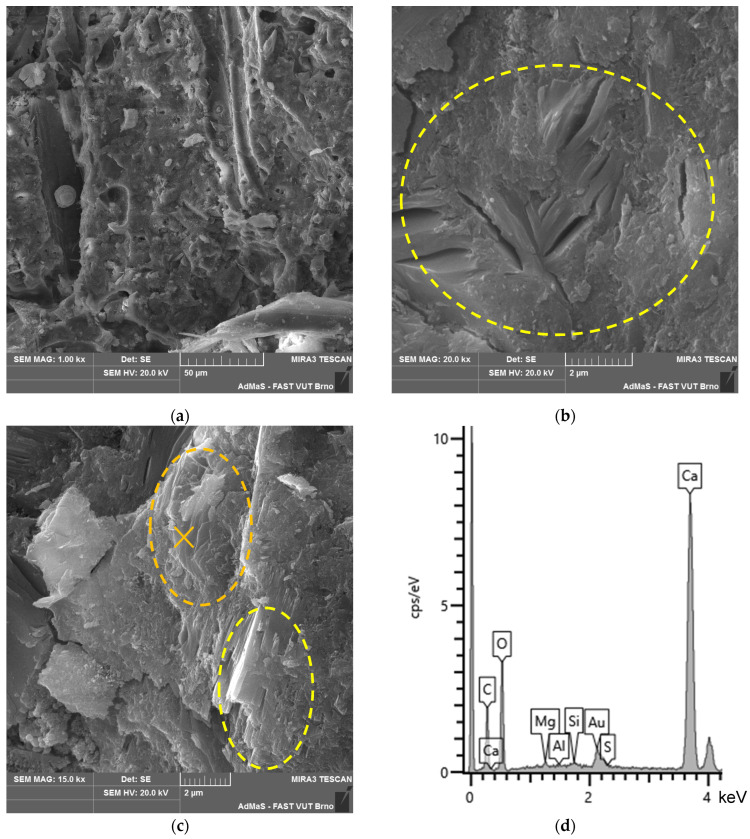
Detail of structure (Tescan MIRA3 XMU scanning electron microscope)—reference boards CBP-R after 730 days ageing in laboratory environment: (**a**) cement matrix, IZT of matrix and chip; (**b**) detail of compact matrix—CASH phases with sulphur (yellow-highlighted); (**c**) matrix CASH phases with sulphur (yellow-highlighted) and portlandite (orange-highlighted); and (**d**) EDX spectrum of portlandite area—orange point “×” (see Figure 19c).

**Figure 20:**
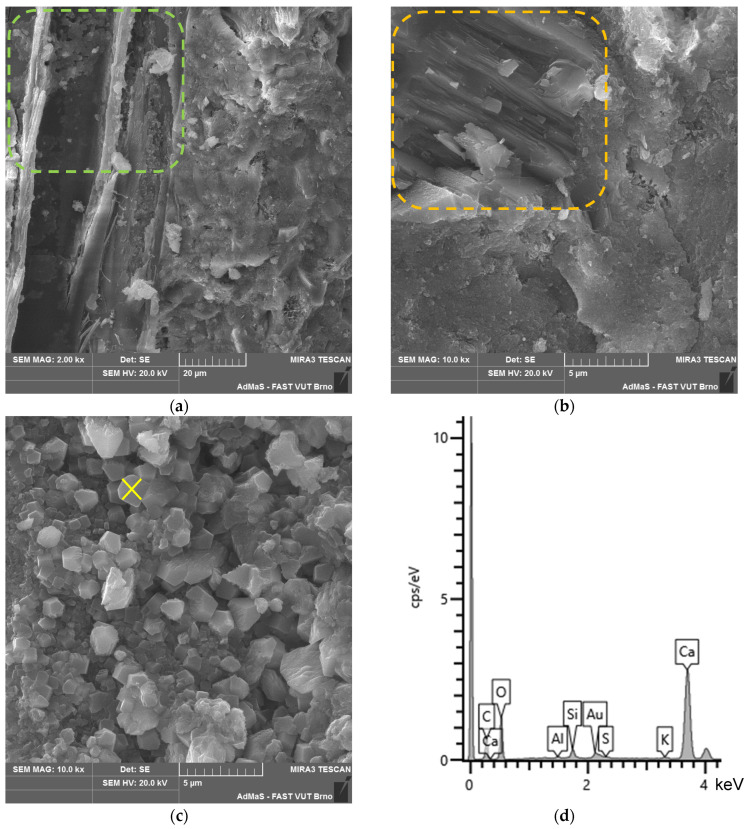
Detail of structure (Tescan MIRA3 XMU scanning electron microscope)—reference boards CBP-R after 730 days exposed to real climatic conditions: (**a**) cement matrix, IZT of matrix and chip, and hydration products in spruce chip (green-highlighted); (**b**) portlandite (orange-highlighted); (**c**) detail of calcite (carbonation product); and (**d**) EDX spectrum of calcite—yellow point “×” (see Figure 20c).

**Figure 21:**
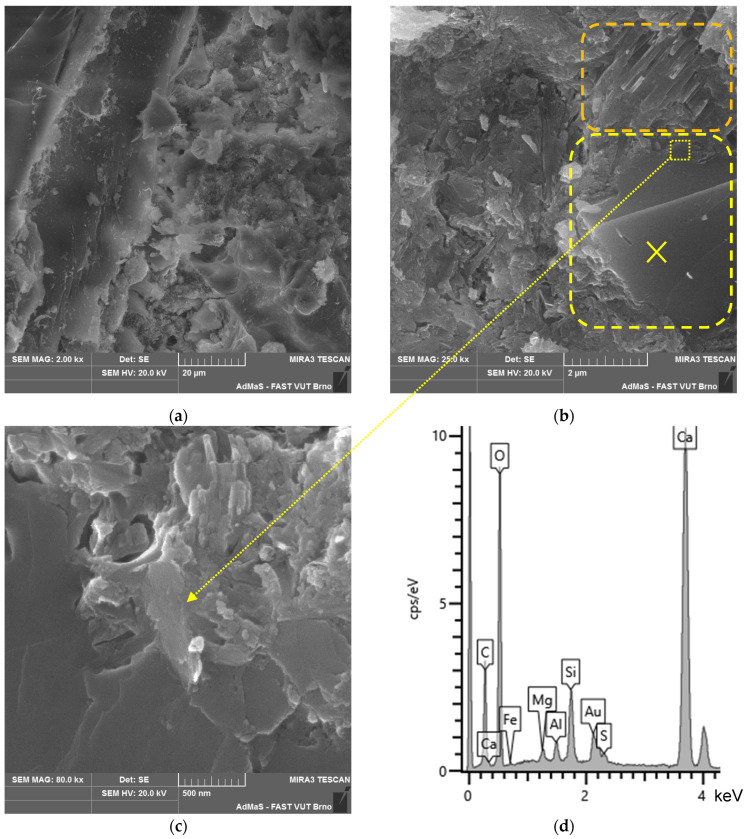
Detail of structure (Tescan MIRA3 XMU scanning electron microscope)—modified boards CBP-L after 730 days ageing in laboratory environment: (**a**) cement matrix, IZT of matrix and chip; (**b**) detail of LS grain in matrix (yellow-highlighted) and portlandite (orange-highlighted); (**c**) ITZ of matrix and LS grain in detail; and (**d**) EDX spectrum of LS grain—yellow point “×” (see Figure 21b).

**Figure 22:**
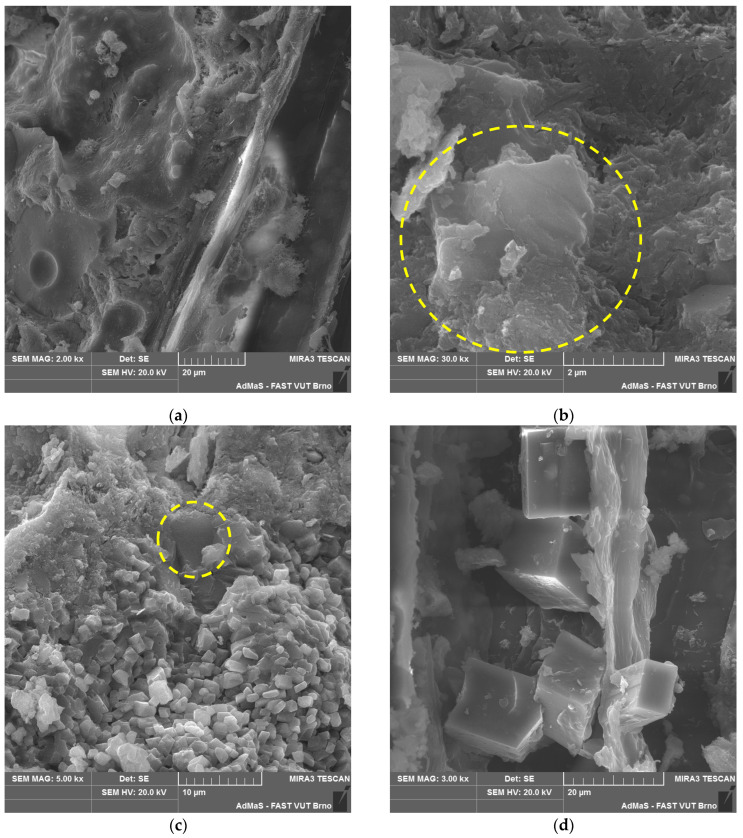
Detail of structure (Tescan MIRA3 XMU scanning electron microscope)—modified boards CBP-L after 730 days ageing in laboratory environment: (**a**) cement matrix, IZT of matrix and chip; (**b**) detail of LS grain in matrix (yellow-highlighted); (**c**) carbonation products and LS grain (yellow-highlighted); and (**d**) detail of carbonation products within spruce chip.

The microstructure of all the analysed types of boards (see Figure 19, Figure 20, Figure 21 and Figure 22) is compact, without apparent defects. The blended cement matrix and chips interact adequately, as evidenced by the very good adhesion of the matrix to the chips. The penetration of hydration products of the matrix, including carbonatation products, into the cellular structure of the spruce chips was also observed.

In the microstructure, CASH phases with bound sulphur ions were identified (see Figure 19b). Analytical techniques such as XRD and DTA did not identify or quantify these phases. These phases are present in all the analysed samples—CBP-R, L, S (stored both in the laboratory and outdoors). The occurrence of these phases is characteristic of the matrix of the analysed CBPs. This phenomenon can be partly explained by the presence of LS. The surface of LS grains provides an ideal substrate for the precipitation of CASH phase nuclei [21].

In CBP-L and CBP-S boards, LS grains were identified. The morphology of LS grains changed over time as the boards aged. In later stages of the exposure under real conditions, a mild reaction of the LS grains surrounded by hydration products of the cement matrix occurred. This reaction of LS grains was locally confirmed by an elemental analysis (see Figure 21b,c). Primarily, however, LS acts more as an inert component. Nevertheless, it synergistically cooperates and is practically perfectly compatible mainly with the CSH or CASH (containing sulphur ions) phases of the matrix. Thaumasite, as mentioned, for example, in [66,67,68], was not identified.

Portlandite was locally identified, and to a greater extent, CSH phases in close proximity to limestone (see Figure 21b). This partially supports the active involvement of limestone during hydration reactions. Limestone, according to studies, improves the course of cement hydration by creating favourable nucleation sites, allowing the growth of CH and CSH [3,17,75]. Additionally, limestone provides additional surface for the nucleation and growth of hydration products [5,76].

Given the differing properties determined for the laboratory and outdoor storage of CBP, the ongoing hydration of cement and the significant reaction of Ca(OH)_2_ with CO_2_ forming CaCO_3_ are crucial (see Figure 20c and Figure 22c). Besides hydration products, carbonate products—quite well-developed CaCO_3_ crystals—were identified in the cellular structure of the spruce chips (see Figure 22d). This phenomenon significantly contributes to achieving a more compact structure of the spruce chips and thus the entire composite. Therefore, CBPs exposed to real climatic conditions were found to have significantly higher, or better, mechanical properties.

## 4. Conclusions

After two years of exposure under real climatic conditions, the microstructure of wood–cement composites (CBPs) is more compact and dense with minimal defects. Significant carbonatation also occurs, contributing to the improvement of CBP properties (bending strength increases from 7.2% to 10.0%). Carbonatation products are visible both in the matrix and in the cellular structure of the spruce chips. The hydration of the CBP matrix occurs over the long term and is nearly complete after two years (marked by a significant decline in the diffraction lines of β-C_2_S). On the basis of the performed experiments, it is possible, in terms of the effect of LS and SCs on CBP behaviour, to conclude the following:

- LS in an amount of 10% has a positive effect on the development of the microstructure, which consequently results in improved mechanical and physical properties of CBPs. The bending strength increased by 4.6% and the modulus of elasticity in bending increased by 4.3% (after 2 years). The hygroscopic behaviour is comparable to the reference CBP;
- LS acts as both an active component in the formation of the cement matrix structure and as an inert microfiller when an inert nature slightly prevails. However, as an inert microfiller, LS is highly compatible and synergistically interacts well with the hydration products of the CBP matrix;
- SCs in an amount of 7% favourably influence the hygroscopic behaviour of CBPs. On the other hand, their smaller size (compared to PC) negatively affects the tensile strength perpendicular to the plane of the board (both in laboratory storage and exposure to real climatic conditions). A decrease of 9.1% in the tensile strength was determined.

Considering further research on the potential activity of LS in the CBP structure, it would be appropriate to give separate attention to its potential influence on the kinetics of reactions with Na_2_SiO_3_ and spruce chips. Future research should verify mechanochemical activation, as discussed, for example, by authors in [77,78]. This method of high-speed milling of either limestone alone or together with cement could significantly contribute to its activation and possibly accelerate the reactions occurring during the formation of the cement matrix structure.

## Figures and Tables

**Table 1:** Composition (mass %) of designed wood–cement material formulas.

Component	CBP-R	CBP-L	CBP-S
Cement CEM II/A-S 42,5 R (CE)	50	45	45
Limestone VMV15-F (LS)	0	5	5
Primary spruce chips (PCs)	18	18	16.7
Secondary spruce chips (SCs)	0	0	1.3
Sodium silicate and aluminium sulphate	2	2	2
Water	30	30	30

**Table 2:** Physical parameters of cement, limestone, and secondary chips.

Parameter	Units	CE	LS	PC	SC
Density	kg/m^3^	3105	2709	-	-
Bulk density	kg/m^3^	-	-	420	1180
Loose bulk density	kg/m^3^	-	-	90	470
Specific surface area	m^2^/kg	408	476	-	-
Water absorption	%	-	-	196.3	51.8

**Table 3:** Chemical composition (mass %) of cement, limestone, and secondary chips.

Component	CE	LS	SC
CaO	59.3	53.6	44.1
CO_2_	-	40.9	-
SiO_2_	18.8	1.3	9.1
Al_2_O_3_	5.6	0.2	11.7
Fe_2_O_3_	3.1	0.1	2.6
Alkali	0.9	0.07	1.6
MgO	1.8	0.5	0.2
Wood content	-	-	30.6

**Table 4:** Mineralogical composition of cement, limestone, and secondary chips.

Material	Phase (Mineral)
CE	Alite, belite, tricalcium aluminate, brownmillerite, gypsum, calcite, periclase, and merwinite
LS	Calcite and aragonite, with traces of magnesite and quartz
SC	CSH phases, portlandite, larnite, and ettringite

**Table 5:** Density coefficient of variation (exposure: /R—laboratory environment and /E—real climatic conditions).

Age (Days)	CBP-R/R (%)	CBP-R/E (%)	CBP-L/R (%)	CBP-L/E (%)	CBP-S/R (%)	CBP-S/E (%)
28	0.80	-	1.07	-	1.22	-
183	0.87	0.63	0.94	1.19	0.97	0.93
365	0.61	0.91	1.15	0.69	0.55	1.07
548	0.92	0.94	0.63	0.76	0.80	0.85
730	0.69	0.65	0.72	0.79	0.66	0.49

**Table 6:** Bending strength coefficient of variation (exposure: /R—laboratory environment and /E—real climatic conditions).

Age (Days)	CBP-R/R (%)	CBP-R/E (%)	CBP-L/R (%)	CBP-L/E (%)	CBP-S/R (%)	CBP-S/E (%)
28	3.99	-	4.99	-	3.33	-
183	2.90	3.67	3.81	5.61	2.68	5.10
365	3.40	3.91	3.25	6.14	1.77	3.28
548	2.71	3.19	3.36	3.52	2.22	3.71
730	2.26	3.40	2.09	1.60	1.52	2.75

**Table 7:** Modulus of elasticity in bending coefficient of variation (exposure: /R—laboratory environment and /E—real climatic conditions).

Age (Days)	CBP-R/R (%)	CBP-R/E (%)	CBP-L/R (%)	CBP-L/E (%)	CBP-S/R (%)	CBP-S/E (%)
28	2.77	-	3.95	-	5.05	-
183	2.54	3.85	5.12	5.16	2.46	4.39
365	4.63	2.40	2.07	3.79	1.47	2.51
548	4.22	3.08	3.92	4.02	4.54	2.39
730	3.64	3.01	2.96	2.72	1.59	3.31

**Table 8:** Transverse tensile strength perpendicular to the plane of the board coefficient of variation (exposure: /R—laboratory environment and /E—real climatic conditions).

Age (Days)	CBP-R/R (%)	CBP-R/E (%)	CBP-L/R (%)	CBP-L/E (%)	CBP-S/R (%)	CBP-S/E (%)
28	3.04	-	5.49	-	7.61	-
183	4.27	1.71	3.09	3.50	3.26	2.79
365	4.80	2.39	4.04	1.82	4.41	2.88
548	3.47	3.43	5.05	2.65	3.27	5.33
730	3.70	2.59	4.86	4.42	4.49	1.82

**Table 9:** Dimensional changes associated with changes in relative humidity—evaluation according to EN 318 (all specimens were stored under laboratory conditions).

Parameter	Units	CBP-R	CBP-L	CBP-S
δl_65,85_	mm/m	0.640	0.620	0.565
δl_65,30_	mm/m	1.275	1.235	1.200
δt_65,85_	%	0.305	0.260	0.245
δt_65,30_	%	0.400	0.360	0.330

**Table 10:** Quantified phase composition of analysed CBP matrix—content of main phases DTA.

Boards/Environment, Age	CSH Phases (%)	Ca(OH)_2_ Portlandite (%)	ΔCa(OH)_2_ Portlandite ΔR–L(S) (%)	CaCO_3_ Calcite (%)
CBP-R/R28	18.05	3.82	-	13.73
CBP-R/R365	20.17	2.60	-	14.02
CBP-R/R730	20.85	2.34	-	16.34
CBP-R/E365	22.24	0.45	-	19.13
CBP-R/E730	21.83	0.08	-	22.84
CBP-L/R28	16.51	3.66	−4.2	15.09
CBP-L/R365	18.29	2.42	−6.9	16.38
CBP-L/R730	19.06	2.11	−9.8	18.95
CBP-L/E365	21.16	0.52	-	22.71
CBP-L/E730	21.97	0.06	-	24.67
CBP-S/R28	16.93	3.85	0.8	16.41
CBP-S/R365	18.14	2.55	−1.9	17.73
CBP-S/R730	19.58	2.17	−7.3	20.09
CBP-S/E365	22.05	0.44	-	22.36
CBP-S/E730	22.34	0.12	-	25.08

## Data Availability

The datasets used and/or analysed during the current study are available from the corresponding author on reasonable request.
